# Hemihypoatrophy as an extraordinary manifestation of linear morphoea

**DOI:** 10.1093/rap/rkac104

**Published:** 2022-12-13

**Authors:** Miguel Mansilla-Polo, Miguel Á Navarro-Mira, Rafael Botella-Estrada

**Affiliations:** Dermatology Department, Hospital Universitario y Politécnico La Fe, Valencia, Spain; Dermatology, IIS La Fe, Valencia, Spain; Dermatology Department, Hospital Universitario y Politécnico La Fe, Valencia, Spain; Dermatology, IIS La Fe, Valencia, Spain; Dermatology Department, Hospital Universitario y Politécnico La Fe, Valencia, Spain; Dermatology, IIS La Fe, Valencia, Spain; Dermatology, Universitat de València, Valencia, Spain

A 41-year-old man attended for evaluation of skin lesions. The lesions began at the age of 8–10 years with alopecia and progressive atrophy in the right leg, which later spread to affect the entire right side of the body (Fig. 1). The patient denied systemic symptoms.

**Figure 1.**
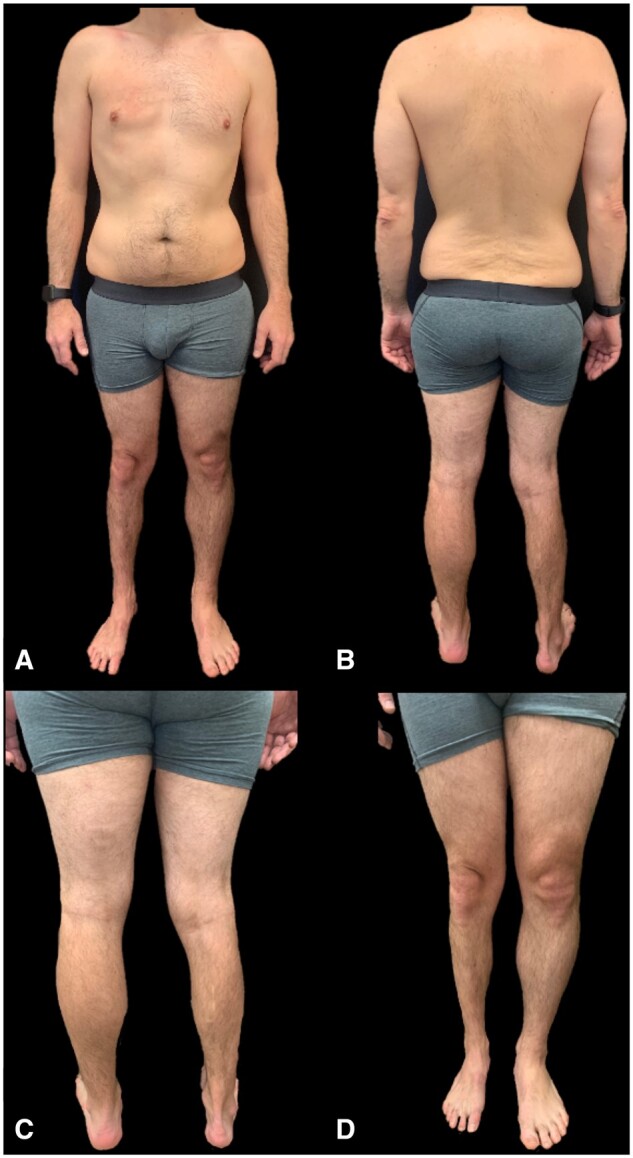
Appearance of the patient at the time of the consultation. Note the marked right hemiatrophy. (**A, B**) Alopecia was especially visible in the upper half of the body and (**C, D**) asymmetry with atrophy in the lower half of the body

The basic analysis was normal and in the autoimmunity study, ANA positivity stood out at a titre of 1/160, with a homogeneous pattern and negative double-stranded anti-DNA (ds-antiDNA). Electromyography, proteinogram and serology (including *Borrelia* and *Rickettsia*) were normal; muscle biopsy showed signs of atrophy and skin biopsy was consistent with late-stage scleroderma. Other causes of hemihypoatrophy, including traumatic, neurologic or other infectious causes such as poliomyelitis virus infection, were ruled out. Given the chronicity of the lesions, no treatment was started.

The hemicorporal presentation of linear morphoea, although extraordinary, has been reported in adults. However, to our knowledge, we present the first case of onset in childhood. Adults typically present with a rapid extension with ascending progression and possible neurological involvement. Persistent atrophy is the norm after stabilization of the condition. Laboratory findings are usually non-specific, although positivity for ANA, antiphospholipid antibodies, ds-antiDNA and eosinophilia have been described. Early treatment can slow the progression of the disease [1, 2].

With this case, we point out a linear morphoea of extraordinary presentation as a corporal hemiatrophy. Recognition of this entity is necessary to avoid late scarring phases, where the therapeutic response is very poor.

## Data Availability

There are no relevant data other than the confidential patient file.

## References

[1] Dharamsi JW , Victor S, Aguwa N et al Morphea in adults and children cohort III: nested case-control study–the clinical significance of autoantibodies in morphea. JAMA Dermatol 2013;149:1159–65. 23925398 10.1001/jamadermatol.2013.4207 PMC4153681

[2] Hassan ML , Saposnik M, Melloni ME et al Morfeas hemicorporales: estudio de siete casos. Rev Arg Reumatol 2013;24:8–14.

